# A questionnaire to measure women’s experiences with pregnancy, birth and postnatal care: instrument development and assessment following a national survey in Norway

**DOI:** 10.1186/s12884-015-0611-3

**Published:** 2015-08-21

**Authors:** Ingeborg S. Sjetne, Hilde H. Iversen, Johanne G. Kjøllesdal

**Affiliations:** Norwegian Knowledge Centre for the Health Services, PO box 7004, St Olavs plass, N-0130 Oslo, Norway

## Abstract

**Background:**

The Norwegian authorities monitor the quality of public health-care services, including from the patients’ perspective. The aim of this paper is to describe the development and psychometric properties of a pregnancy- and maternity-care patients’ experiences questionnaire (PreMaPEQ).

**Methods:**

The PreMaPEQ and data collection procedures were developed based on a literature review, reference group activities, user interviews, cognitive interviews and a pilot test. The PreMaPEQ was then used in a national survey that included a retest distribution. The participants were identified from the hospital records where the birth took place. The invitation to take part was sent by mail and the questionnaire was distributed in electronic (i.e. via the Internet) and (subsequently) paper forms. The completed questionnaires were assessed using descriptive statistics, explorative factor analyses, psychometric measures and confirmatory factor analysis (CFA).

**Results:**

The PreMaPEQ response rate for the national sample was 56.6 % (*N* = 4904), and retest data were provided by 123 women. Statistics and theoretical considerations were used to construct 16 scales, covering the following 4 phases of the care: pregnancy control (4 scales), the delivery (3 scales), the postnatal hospital stay (5 scales) and the services in the public health clinic (4 scales). All scales had a Cronbach’s α of >0.7, and all but three scales had an intraclass correlation coefficient for test-retest stability of >0.700. CFA revealed a satisfactory fit between the questionnaire data and the model, with a four-factor solution of the care experiences with pregnancy, birth and postnatal care. CFA provided support for the suggested structures, and demonstrated that the first-order factors are indicators of a second-order factor.

**Conclusion:**

The PreMaPEQ appears to be an acceptable, valid and reliable tool for collecting women’s experiences of the whole course of maternity care in health systems that have features in common with the Norwegian health system.

**Electronic supplementary material:**

The online version of this article (doi:10.1186/s12884-015-0611-3) contains supplementary material, which is available to authorized users.

## Background

Collection of patient-reported outcomes, including patient experiences, is an important aspect of evaluations of health services. According to an international review, several countries have programs for monitoring the quality of health care using surveys that inquire into the experiences of patients and other health-care users. These surveys call for descriptions of mainly non-technical aspects of the health-care services and may involve different target populations, such as the general population, broad groups of service users, or patients with specific conditions [1]. The users of the results also vary among the different national programs between health authorities, health-care managers at different levels, health insurers, providers, potential service users, and researchers. Depending on the survey design, the results can be used to monitor health-system performance and/or inform quality improvement efforts at the level of service delivery.

In Norway, the responsibility to conduct surveys of those who use health services is assigned to the Norwegian Knowledge Centre for the Health Services (NOKC), which is a public organization that operates under the Norwegian Directorate of Health. NOKC has developed a variety of data collection tools and surveyed a range of target groups. The explicit purpose of these surveys is fourfold: social legitimacy and control, business control, professional quality improvement, and to inform choices made by patients. In 2009, the Ministry of Health and Care Services issued a white paper entitled “A happy event. About a comprehensive pregnancy, birth and postnatal care” [2], in which the Ministry commissioned a national user survey of women who had recently given birth and their partners. The whole course of the health-care event (i.e., from pregnancy to postnatal care) was to be included, with special attention paid to immigrant women.

The purpose of this paper was to describe the development and the psychometric properties of the pregnancy- and maternity-care patients’ experiences questionnaire (PreMaPEQ).

## Methods

### Instrument development

Development of the PreMaPEQ followed an established procedure used previously by NOKC for developing data-collection tools and routines for conducting surveys in new target groups. The initial literature search in 2009 did not identify any instruments that met the specifications of this study [3], but did provide some information about relevant topics. Hence, a development process was commenced [4], the first step of which was to set up a reference group including service users, authorities and clinicians. The purpose of this group was to collect comments and views during the development process from various stakeholders representing important expertise. The group met three times and discussed questionnaire contents and inclusion criteria. The second step was to explore what is important for people in this situation by interviewing women and their partners with recent experiences with the services. The interviews were semi-structured, individual, and face to face, and the answers underwent conventional content analysis [5]. The interviewees varied with regard to age, parity and ethnicity, and the information they provided was highly consistent with findings from the literature search. The third step was to construct the questionnaire itself. Four sections were constructed, each with a specific colour to reflect the different phases of the health-care course; that is, pregnancy control with green headings, birth with red headings, postnatal hospital stay with orange headings, and finally follow-up in the community health clinic (helsestasjon in Norwegian) with violet headings. The items (i.e. questions) in the questionnaire asked whether desirable properties or behaviours were present, with the intention of collecting a description that was concrete and factual rather than being judgmental [6]. Among the many possible response formats [7] most of the questions were answered on the following five-point ordered response scale: 1 = not at all, 2 = to a small extent, 3 = to some extent, 4 = to a large extent and 5 = to a very large extent. The alternative answer of “not applicable” (NA) was also allowed where it was important to discriminate between user missing and answers that were skipped because the respondent had not used the service in question. This five-point scale performed better that a ten-point scale in a previous study, and was considered more suitable for assessing patient experiences [8]. Consequently, the former has been chosen to be consistently applied in NOKC’s surveys, making it possible to compare over time and, to some extent between different groups of health care users. In the fourth step, the questionnaire was administered to 18 women, who were then interviewed to evaluate whether its structure, questions and response formats were performing as intended [9]. The fifth step was a pilot test that was conducted in a university hospital, in which all women aged 16 years old or older who gave birth over a 2-month period were included (births in which death occurred were excluded). The pilot was conducted as a small version of the large survey to come, to provide an opportunity to detect flaws and weaknesses in the study in time to correct them [10]. This pilot test also provided the opportunity to test and compare the efficiency and cost of two different data-collection routines, for which reason the sample was randomly divided into two groups. The sixth step involved revising the questionnaire in accordance with the findings of the pilot test, and then evaluating it by administering it to 13 women followed by interviewing them to evaluate that final version [11]. Technical revisions were made to capture the various combinations of services the women had used, to cover the diversity in a best possible way. The last question asked whether the respondent would be willing to answer a new questionnaire after a short interval so that the stability of the results could be tested (i.e. test-retest).

The printed version of the questionnaire consisted of 145 items on 16 pages. The contents were generic, so that unusual but still relatively frequent events were left out, such as unplanned births at home or during transport. The women may have used many different combinations of services, both public and private, and not all variations could be captured by the questionnaire.

The final questionnaire was translated into English for facilitating responses from immigrants. The translations were carried out according to recommended procedures [12]. We had no knowledge of the linguistic abilities of the potential respondents, so the invitation was to all otherwise eligible women. See Additional file 1 for the printed version in English.

The same procedure was used to construct a questionnaire targeting the women’s partners; however, that questionnaire was not assessed in this paper.

### Setting

The focus of this nationwide survey was the entire care course from the first visit in prenatal services, through birth and postnatal care in birth institutions, and finally to the follow-up in community health clinics. The financing and delivery of prenatal monitoring and postnatal follow-up in health clinics is the responsibility of the municipality of residence of the individual women, while the birth institution—be it in a large hospital or a local maternity clinic—is the responsibility of the state, via the hospital trusts.

### Sampling

Women who gave birth in the last quarter of 2011 in a Norwegian institution and who were 16 years old or older were included. Experiences from previous surveys led to the requirement of samples with 400 potential respondents from each hospital. The women to be included were drawn randomly from institutions with a large number of births (more than 400 during the inclusion period), and women were included consecutively from institutions with less than 400 births. The Medical Birth Registry conducted the sampling routine. Before any list of names and addresses were transferred to the Knowledge Centre, information from the National Population Registry Office was collected and any birth in which either the woman or child had died was excluded.

### Data collection

Potential respondents to the questionnaire were first contacted by mail about 17 weeks after the birth: they were sent a letter with information about the survey and an invitation to participate via the Internet, including a specific username and password. Two reminders were sent to non-respondents, both of which included a printed version of the questionnaire in addition to a username and password.

The names and addresses were deleted when all of the mailings were completed, and the questionnaire data were supplemented by clinical information from the Medical Birth Registry. Statistics Norway provided data regarding the country of origin for the women included in the survey and their parents.

Informed consent was considered expressed when the women had received the mailed information and submitted their response. The Regional Committee for Medical and Health Research Ethics (REK sør-øst D) approved the study.

### Data analyses

SPSS software (version 15.0, SPSS, Chicago, IL, USA) was used to analyse the sample and variables, and for exploratory factor analysis (EFA).

Questionnaire items regarding experiences were entered into principal axis factoring analyses according to phase; that is, items pertaining to pregnancy control, birth, postnatal hospital stay and the public health clinic. EFA results were interpreted as supportive of a factor if loadings exceeded 0.40 and there were no cross-loadings. Some correlations between the factors were assumed, and the oblique rotation method of analysis was chosen [13].

In cases where the resulting factors after EFA included diverse phenomena, the factors were split and the items grouped according to the structure and process categories in Donabedian’s framework [14] or to ensure that the survey results reflected recognizable elements of care delivery. For example splitting structure (resources and organization) and process (personal relationships) or splitting information about women’s health and information about the child. We believe that this will render the survey results more useful for local improvement purposes. Items that were not included in a factor, due to poor factor loadings or high missing rate, were reported as single items. The final outline of the questionnaire is shown in Table 1.

**Table 1:**
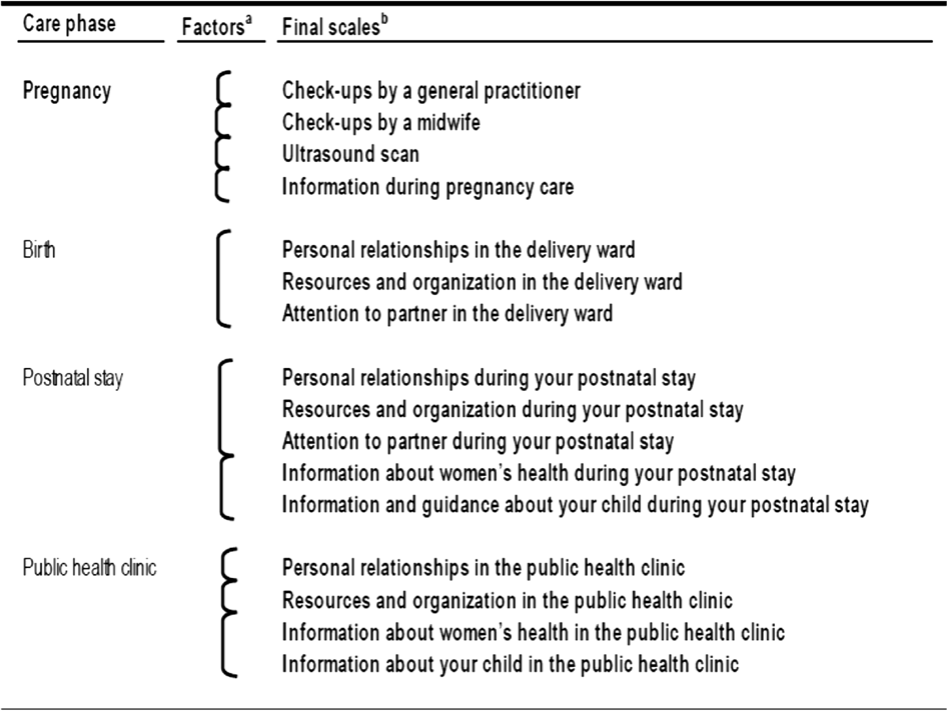
Outline of the questionnaire. Care phases, questionnaire factors and final scales (indexes)

^a^Scales as suggested by EFA results

^b^Scales (indexes) after splitting factors by structure- and process categories or specific contents

Most single items were scored on a scale of 1–5, and the index scores were transformed linearly to a scale of 0–100.

Impression of potential differences between respondents and non-respondents were obtained by comparing those who responded to the first contact with those who responded after two reminders. In wave analyses such as this, the latter—which are the most difficult to obtain—are considered proxies for non-respondents [15].

The LISREL analysis program (version 8.8) was used to test the goodness of fit of the models [16] by confirmatory factor analysis (CFA), and was applied to further test the relationships between the manifest variables and their underlying latent constructs. The 16 scales were constructed on the basis of a combination of the theoretical structure and process categories in Donabedian’s framework, our EFA results, and to some extent the specific content of the items. Therefore the scales were not only data-driven, but also founded on a theoretical understanding underpinning the analyses. Accordingly, the CFA did not test a model based solely on the correlation of the test items but whether the measures are consistent with our understanding of the nature of the construct. Consequently, the objective was to test whether the data fitted our hypothesized model based on theory and analytic research.

It was hypothesized that there was a second-order factor structure for the instrument, with experiences with pregnancy control, the birth, the postnatal hospital stay, and the public health clinic as the lower-order factors, and care experiences in pregnancy-, birth- and postnatal care as the higher-order factors. In CFA there are two types of latent variables; endogenous and exogenous. Exogenous variables always act as independent variables, endogenous on the other hand, are variables that are influenced by other variables in the model.

Various fit indexes were used, including the root-mean-square error of approximation (RMSEA), goodness-of-fit index (GFI), comparative fit index (CFI) and incremental fit index (IFI). An RMSEA of ≤0.05 and GFI and CFI values of ≥0.90 are generally taken to indicate a good fit. IFI values range from 0 to 1, with larger values indicating a better goodness of fit.

## Results

Among the 8670 eligible women in the sample, 4904 returned completed questionnaires, giving a 56.6 % response rate. Every fifth woman in the total sample responded after the first contact, and responding via the Internet was their only option (see Table 2). Among those who received one or two reminders (i.e. those who could choose to fill in a paper questionnaire instead of answering via the Internet), the majority opted to answer on paper (23 %) rather than via the Internet (12 %). The median completion time was 20 min for the women responding via Internet (inter quartile range 15–31).

**Table 2:** Sample response mode according to level of contact (*N* = 8670)

	First contact^a^	First reminder^b^	Second reminder^b^
Response via Internet	21 %	7 %	5 %
Response on paper	NA	12 %	11 %

^a^Responses via Internet only

^b^Responses via Internet or on paper

*NA* Not applicable

As listed in Table 3, the respondent and non-respondent groups differed with regard to age, parity, and ethnicity.

**Table 3:** Sample descriptives

	Non-respondents		Respondents		Total		
	*N*	%	*N*	%	*N*	%	*p* for differences^a^
*Age (years)*							<0.001
≤ 25	1026	27.2	957	19.5	1983	22.9	
> 25 ≤ 28	702	18.6	904	18.4	1606	18.5	
> 28 ≤ 31	691	18.3	1078	22.0	1769	20.4	
> 31 ≤ 35	789	21.0	1161	23.7	1950	22.5	
> 35	558	14.8	804	16.4	1362	15.7	
*Parity*							<0.000
First	1416	37.6	2344	47.8	3760	43.4	
Second	1323	35.1	1662	33.9	2985	34.4	
Third	697	18.5	670	13.7	1367	15.8	
Fourth or more	330	8.8	228	4.6	558	6.4	
*Ethnicity*							
Non-Norwegian, non-Western	789	21.0	426	8.7	1215	14.0	<0.000
Non-Norwegian, Western	378	10.0	450	9.2	828	9.6	<0.000
*The birth*							
Multiple birth	60	1.6	69	1.4	129	1.5	0.476
Epidural or spinal anaesthesia (excluding caesarean)	1075	28.5	1324	27.0	2399	27.7	0.115
*Mode of delivery*							0.083
Vaginal delivery	3144	83.7	3999	82.2	7143	82.9	
Emergency caesarean	384	10.2	571	11.7	955	11.1	
Planned caesarean	230	6.1	294	6.0	524	6.1	
*Geographic region*							0.460
Southeast	2039	54.1	2639	53.8	4678	54.0	
West	664	17.6	901	18.4	1565	18.1	
Central	580	15.4	781	15.9	1361	15.7	
North	483	12.8	583	11.9	1066	12.3	
*Institution size (no. of births per year)*							0.111
< 49 + other/unspecified	30	0.8	41	0.8	71	0.8	
50–499	601	16.0	701	14.3	1302	15.0	
500–1499	920	24.4	1194	24.3	2114	24.4	
1500–2999	1232	32.7	1652	33.7	2884	33.3	
> 3000	983	26.1	1316	26.8	2299	26.5	
*Marital status* ^*b*^							
Married			2131	44.6			
Cohabiting			2502	52.3			
Neither married nor cohabiting			149	3.1			
*Education* ^*b*^							
Primary school			205	4.3			
High school			1393	29.1			
University undergraduate			1864	38.9			
University postgraduate			1327	27.7			
*Main activity when not on maternity leave* ^*b*^							
Working			3839	80.1			
Sick leave or welfare allowances			129	2.7			
Education			350	7.3			
Homemaking			126	2.6			
Unemployed			279	5.8			
Other			70	1.5			
*Self-rated health* ^*b*^							
Poor			52	1.1			
Fair			312	6.5			
Good			1366	28.6			
Very good			2053	42.9			
Excellent			1001	20.9			

^a^ *χ* ^2^ test

^b^Data from respondents only

EFA yielded six factors describing experiences with pregnancy care. One factor pertaining to cooperation, for example between the community midwife and the hospital, was disqualified by a large number of “Don’t know” responses. Another factor regarding incorrect treatment and conflicting information was disqualified because it failed the internal consistency tests [7]. Hence, the pregnancy-care phase was described by four scales in the final instrument (Table 4).

**Table 4:** Scale descriptives and psychometric properties

Scale and items	Item number	Mean^a^	SD	Non-response/“Don’t know”/NA (%)	Scale internal consistency (Cronbach’s α)	Item-total correlation coefficient	Test-retest reliability (ICC, *N* = 123)
*Check-ups by a general practitioner*		*76.3*	*21.6*	*20.3*	*0.926*		*0.833*
Did the general practitioner treat you politely and with respect?	21	4.3	0.8			0.796	
Did the general practitioner spend enough time at the visits?	22	3.8	1.1			0.774	
Did you find that the general practitioner was open to your questions?	23	4.1	1.0			0.879	
Did you find that the general practitioner cared about you?	24	4.0	1.0			0.867	
Did you have confidence in the general practitioner’s professional competence?	25	4.0	1.0			0.755	
*Check-ups by a midwife*		*88.0*	*15.5*	*14.3*	*0.918*		*0.662*
Did the midwife treat you politely and with respect?	12	4.6	0.6			0.790	
Did the midwife spend enough time at the visits?	13	4.5	0.7			0.725	
Did you find that the midwife was open to your questions?	14	4.6	0.7			0.843	
Did you find that the midwife cared about you?	15	4.5	0.8			0.846	
Did you have confidence in the midwife’s professional competence?	16	4.5	0.8			0.763	
*Ultrasound scan*		*78.3*	*20.8*	*4.2*	*0.828*		*0.716*
Did you receive sufficient information concerning the ultrasound scan?	35	4.1	0.8			0.709	
Were you happy with the midwife/doctor who performed the ultrasound scan?	36	4.2	0.9			0.709	
*Information during pregnancy care*		*66.6*	*21.1*	*3.5*	*0.872*		*0.814*
Did you receive sufficient information about the following?:							
Your physical health during the pregnancy	26	4.1	0.9			0.559	
Possible mood changes during the pregnancy	27	3.6	1.1			0.692	
How the baby was developing	28	4.0	0.9			0.638	
What you could expect regarding the birth	29	3.6	1.1			0.775	
Options for pain relief during the birth	30	3.5	1.2			0.725	
Post-natal period (e.g. breastfeeding, nutrition, care for the child)	31	3.1	1.3			0.679	
*Personal relationships in the delivery ward*		*81.0*	*18.7*	*10.3*	*0.906*		*0.778*
Were you treated politely and with respect by the health-care personnel at the delivery ward?	52	4.3	0.8			0.770	
Did you find that the health-care personnel were open to your questions?	54	4.2	0.8			0.839	
Did you find that the health-care personnel cared about you?	55	4.2	0.8			0.837	
*Attention to partner in the delivery ward*		*84.2*	*18.0*	*11.5*	*0.727*		*0.783*
Was your partner received well by the health-care personnel at the delivery ward?	65	4.3	0.8			0.573	
Were things arranged so that your partner could be present if you both so wished?	66	4.4	0.8			0.573	
*Resources and organization in the delivery ward*		*76.0*	*17.7*	*10.4*	*0.867*		*0.874*
Were you received well when you arrived at the delivery ward?	51	4.2	0.8			0.561	
Did the health-care personnel have time for you when you needed it?	53	4.1	0.9			0.715	
Did you have confidence in the health-care personnel’s competence?	56	4.3	0.8			0.706	
Did you receive sufficient information during your stay at the delivery ward?	57	3.9	0.9			0.715	
Did you find that the services you received during your stay at the delivery ward were well-organized?	59	3.9	0.9			0.734	
Did you find that the health-care personnel cooperated well during the birth?	60	4.2	0.9			0.704	
Did you receive information about who had the main responsibility for you?	61	3.6	1.3			0.485	
*Personal relationships during your postnatal stay*		*76.8*	*20.2*	*5.8*	*0.912*		*0.844*
Were you treated politely and with respect by the health-care personnel during your postnatal stay?	75	4.1	0.8			0.798	
Did you find that the health-care personnel were open to your questions?	77	4.0	0.9			0.842	
Did you find that the health-care personnel cared about you and your child?	78	4.1	0.9			0.836	
*Attention to partner during your postnatal stay*		*74.3*	*24.0*	*7.7*	*0.793*		*0.828*
Was your partner received well by the health-care personnel during your postnatal stay?	91	4.0	0.9			0.670	
Were things arranged so that your partner could be present if you both so wished?	92	3.9	1.2			0.670	
*Information about women’s health during your postnatal stay*		*58.3*	*26.2*	*6.6*	*0.825*		*0.663*
Did you receive sufficient information about the following?:							
Your physical health after giving birth	80	3.5	1.1			0.705	
Any possible mood changes after giving birth	81	3.2	1.2			0.705	
*Information and guidance about your child during your postnatal stay*		*67.2*	*24.1*	*7.7*	*0.901*		*0.826*
Did you receive sufficient information about the following?:							
Breastfeeding and other ways of feeding the child	82	3.8	1.0			0.755	
Child care	83	3.6	1.1			0.799	
Did you receive sufficient guidance on the following?:							
Breastfeeding and other ways of feeding the child	85	3.8	1.1			0.784	
Child care	86	3.6	1.1			0.782	
*Resources and organization during your postnatal stay*		*65.6*	*20.9*	*6.0*	*0.875*		*0.890*
Did the health-care personnel have time for you when you needed it?	76	3.9	1.0			0.748	
Did you have confidence in the health-care personnel’s professional competence?	79	4.1	0.9			0.679	
Did you find that the services you received during your postnatal stay were well organized?	87	3.7	1.0	7.6		0.802	
Did you find that the health-care personnel cooperated well during your postnatal stay?	88	3.7	1.0			0.817	
Did you receive information about who had the main responsibility for you?	89	2.7	1.3			0.536	
Were things arranged so that you could get enough peace and rest?	90	3.6	1.2			0.594	
*Personal relationships in the public health clinic*		*76.8*	*20.2*	*2.7*	*0.887*		*0.748*
Are you treated politely and with respect by the staff?	114	4.4	0.7			0.736	
Do you find that the staff are open to your questions?	116	4.4	0.7			0.798	
Do you find that the staff care about you and your child?	117	4.4	0.7			0.804	
*Information about women’s health in the public health clinic*		*56.7*	*25.9*	*3.5*	*0.828*		*0.697*
Did you receive sufficient information about the following?:							
Your physical health after giving birth	120	3.1	1.1			0.706	
Possible mood changes after giving birth	121	3.4	1.1			0.706	
*Information about your child in the public health clinic*		*75.3*	*17.9*	*2.8*	*0.831*		*0.748*
Did you receive sufficient information about the following?:							
The child’s development and health	122	4.2	0.7			0.706	
Vaccines for the child	123	4.3	0.7			0.631	
Breastfeeding and other ways of feeding the child	124	4.0	0.9			0.716	
Child care	125	3.6	1.1			0.648	
*Resources and organization in the public health clinic*		*79.9*	*16.3*	*2.6*	*0.768*		*0.807*
Do the staff spend enough time at the check-ups?	115	4.4	0.7			0.577	
Do you have confidence in the professional competence of the staff?	118	4.2	0.9			0.632	
Do you find that the care you receive at the health clinic is well organized?	127	4.1	0.8			0.607	

^a^High scores represent positive descriptions; range of 1–5 for individual items and 0–100 for scales

*ICC* Intraclass correlation coefficient

Twelve items produced one factor covering diverse aspects of birth care, and we chose to split that factor. Furthermore, we decided that two items about how the partner was taken care of should form a single index. Ten items were regrouped based on Donabedian’s framework to cover interpersonal relationships (three items), and structure in the form of organization, material and human resources (seven items). One factor containing three items pertaining to incorrect treatment and conflicting information during the birth was left out because it failed internal consistency tests.

The fourth column in Table 4 lists the prevalence of omitted or NA responses. The prevalence rates of such responses were highest for check-ups by a general practitioner and by a midwife, at just above 20 and 14 %, respectively. This reflects that not all of the women had their check-ups completed by both health-care personnel. The prevalence of missing or NA responses with regard to experiences in the delivery room was 10–11 %. We believe that this was attributable to unclear wording of a filter question. Incorrect answers to this filter question led to the items comprising this index being withheld from respondents who used the Internet option to complete the questionnaire. For the remaining items, the proportion of respondents who gave the NA response was highest for questions that would be largely irrelevant for all respondents other than first-time mothers.

The mean proportion of omitted answers was 2 % (range = 0.8–3.8 %) among the items included in the indexes [17]. There was no tendency for this proportion to increase from the start to the end of the questionnaire.

Comparison between those who responded to the first contact and those who responded to the second reminder revealed statistically significant differences on 7 of the 16 indexes; the scores for the responders to the first contact were the most positive on all but one index, with a mean difference of 1.5 points (range = 1.3–3.7).

The internal consistency of the constructed scales, as measured by Cronbach’s α, varied from 0.727 to 0.926 (median = 0.870). The test-retest stability of the scales, as measured by the intraclass correlation coefficient, varied from 0.662 to 0.890 (median = 0.795) between the scales.

The measurement model for the endogenous latent variables stipulates the relationship between the endogenous latent variables (η) and the corresponding manifest variables (γ). In this model the manifest variables included latent variables. The questionnaire was comprehensive, but it was not possible to include all of the observed variables or items. The measurement of experiences with pregnancy control, the delivery, the postnatal hospital stay, and the public health clinic comprised four, three, five and four manifest variables, respectively. The structural part of the model stipulates the relationship among the endogenous latent variables (η) and the exogenous latent variable (ζ).

**Fig. 1:**
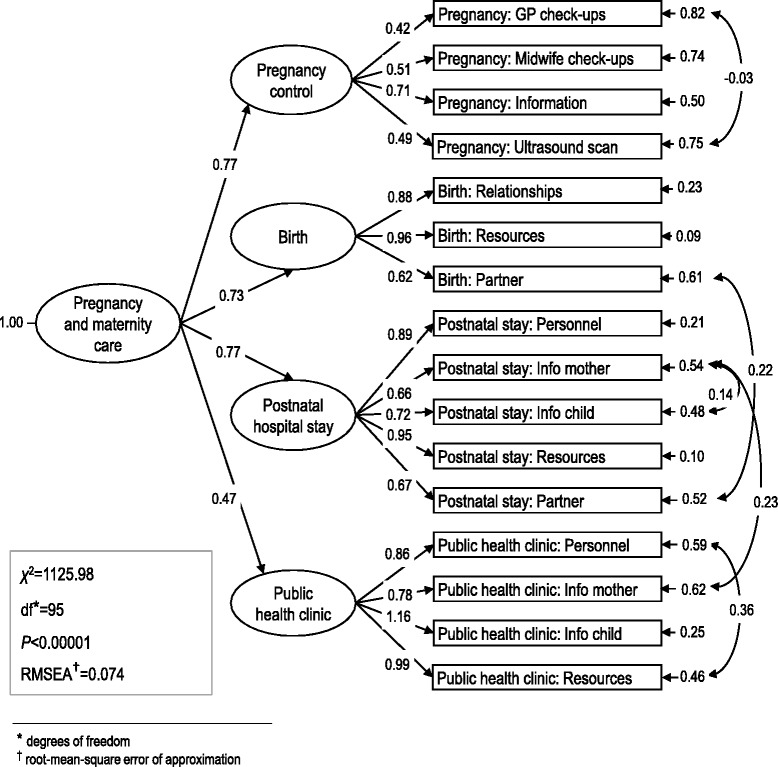
Confirmatory factor analysis model (Full text annotations of the latent variables in Table 4)

The four-factor solution of the care experiences for pregnancy, birth and postnatal services in the PreMaPEQ was tested, and revealed that there was a satisfactory model fit to the data (*χ*^2^ = 1125.98, *p* < 0.001, degrees of freedom = 95, RMSEA = 0.074, GFI = 0.93, CFI = 0.90 and IFI = 0.97). The results are shown in Fig. 1. The exogenous latent variable was labelled pregnancy and maternity care, and the four endogenous latent variables were labelled experiences with pregnancy control, the birth, the postnatal hospital stay, and the public health clinic, introducing a second-order analysis. Experiences with pregnancy control (γ = 0.77) and postnatal care (γ = 0.77) had the strongest relationships with the exogenous latent variable, but experiences with the delivery (γ = 0.73) and the public health clinic (γ = 0.47) were also strongly associated with the exogenous latent variable.

## Discussion

This study developed and assessed the properties of a tool, the PreMaPEQ, for measuring user experiences with health care through the phases of pregnancy and childbirth. The instrument development procedure was designed to ensure good content validity, and the assessment indicated that the questionnaire has good reliability. The PreMaPEQ can be used either as a whole or in parts that are adapted to the service in question. The English version is ready to use, since several measures were taken in the translation process to ensure consistency with the Norwegian questionnaire.

There are some limitations to this study. We would have preferred a higher response rate than 56.6 %. However, this compares well with the rate we have achieved in recent surveys among somatic patients in Norway [18]. The low percentage of omitted answers suggests good acceptability, and the lack of increase in omitted answers indicates that the length of the questionnaire does not tire the respondents. Few respondents used the NA response option when this was presented, which indicates that the topics of the questionnaire are relevant to a large majority of the population.

The procedures for assessing aspects of reliability produced coefficients that for the majority of the scales were above the recommended 0.700 limit [7].

It can be argued that a questionnaire with 145 questions is too long, producing a response burden that is too great. The length was a consequence of including all of the phases applicable to this specific field of care. It is a political ambition to produce services of the same quality for all Norwegian citizens. The use of a centralized national survey that is uniform across the different phases of care and geographical regions is more likely to yield data that are comparable.

The purpose of the literature review previous to the national survey was to identify and describe relevant national surveys and validated instruments with a primary focus on user experiences and satisfaction with different parts of maternity care [3]. The review showed that there are variations in approach and methods for both national surveys and validated instruments regarding how long after the birth women are asked to complete the questionnaires, from ten days to 14 months [19–21]. The women received the questionnaire about 17 weeks after the birth. This relatively long period was necessary since we also wanted their information about the postnatal contacts they had with the public health clinic. Although this long period may have caused some recall bias, studies have indicated that information about major life events, such as pregnancy and childbirth, are more easily retrieved compared to information about fluctuating phenomena [22].

We considered it important to include experiences from the public health clinics in the national survey, but acknowledge that precision of reported data about past experiences will always be threatened by the limitations of the respondents memory and the influence of exposure status on the recalling process. As pointed out by Bat-Erdene and colleagues, maternally reported data about the events occurring during labour and delivery are widely used, but the validity of this data is rarely confirmed [23]. However, their study showed that maternal recall at four months post-partum of important events that occurred during labour and delivery is excellent.

The results of the EFAs and tests of internal consistency provided empirical support for the multi-item scales, and confirmed that experiences with the care received during pregnancy, birth and postnatal care are multidimensional concepts. The confirmatory factor analysis provided support for the structures suggested by the EFAs, and demonstrated that the first-order factors are indicators of a second-order factor.

## Conclusions

We conclude that the PreMaPEQ is a valid, reliable and acceptable instrument for collecting women’s experiences of the entire course of maternity care in health systems with features in common with the Norwegian health system.

## Additional file

Additional file 1:
**English version of the printed questionnaire.** (PDF 1155 kb)
