# Supplementary material for: A questionnaire to measure women’s experiences with pregnancy, birth and postnatal care: instrument development and assessment following a national survey in Norway
Source: BMC Pregnancy Childbirth. 2015 Aug 21;15:182. doi: 10.1186/s12884-015-0611-3 (PMC4546178; doi:10.1186/s12884-015-0611-3)
Supplement: Additional file 1: — English version of the printed questionnaire. (PDF 1155 kb) [file 12884_2015_611_MOESM1_ESM.pdf]

kunnskapssenteret
Nasjonalt kunnskapssenter for helsetjenesten

2011/ 2012

To you who have given birth recently!

Questionnaire about experiences with care during pregnancy, birth and the post-natal period

The purpose of this questionnaire is to learn about how women who have recently given birth experience the public health care services offered in Norway during the pregnancy, childbirth and post-natal period. The ultimate goal is to improve the quality of the services.

You can also participate by accessing the survey at www.pasopp.com and enter the username and password printed in the information letter.

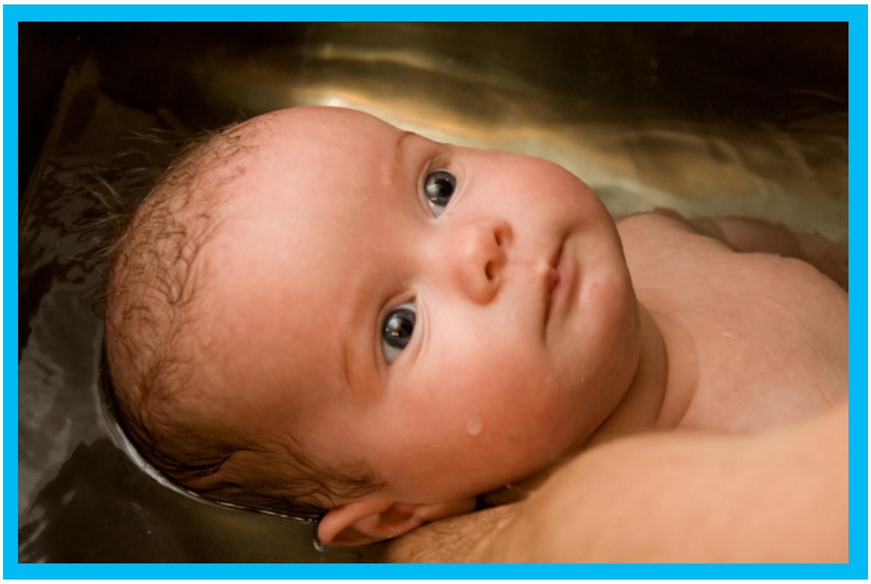

Your experiences are important and we hope you take the time to answer our questions!

If you have given birth earlier, we ask that you consider the care you received in connection with your *most recent* pregnancy, birth and post-natal period.

PART A: THE PREGNANCY

The public health services offer pregnancy check-ups by a midwife and/or general practitioner plus one routine ultrasound scan. Some pay for additional private services, but these will not be considered here.

1. Did you use public health services during pregnancy, for instance pregnancy check-ups by a midwife/general practitioner or routine ultrasound scan (which is usually done at a hospital)?
   - ☐ Yes → go to question 2 below
   - ☐ No → go to question 44 on page 6

	Midwife at health clinic (helse stasjon)	General practitioner	Doctor at health clinic (helse stasjon)	Midwife at hospital	Doctor at hospital	Other	Not applicable
2. From whom did you receive your check-ups during pregancy? *You may select more than one answer*	☐	☐	☐	☐	☐	☐	☐

	Less than 8 weeks	8-12 weeks	13-20 weeks	21-30 weeks	More than 30 weeks	Not sure	Not applicable
3. How many weeks into pregnancy were you when you had the first pregnancy check-up by a midwife or doctor?	☐	☐	☐	☐	☐	☐	☐

	1 - 4	5 - 9	10 - 16	17 or more	Not sure	Not applicable
4. How many pregnancy check-ups did you have in total from a midwife/doctor?	☐	☐	☐	☐	☐	☐

	Yes	No, too few	No, too many	Not applicable
5. Do you think this was an appropriate number of check-ups?	☐	☐	☐	☐

	Yes	No	Not sure	Not applicable
6. Were you able to have your pregnancy check-ups by a midwife if you wanted to?	☐	☐	☐	☐
7. Were you able to have your pregnancy check-ups by a general practitioner if you wanted to?	☐	☐	☐	☐

8. Did you have pregnancy check-ups by a midwife?
   - ☐ Yes → go to question 9 below
   - ☐ No → go to question 17 on page 3

Pregnancy check-ups by a midwife

	1	2	3	4 or more
9. From how many different midwives did you receive pregnancy check-ups?	☐	☐	☐	☐

	Not at all	To a small extent	To some extent	To a large extent	To a very large extent	Not applicable
10. Was it easy to get an appointment with a midwife if you wanted to?	☐	☐	☐	☐	☐	☐
11. Was it important to you to get appointments from the same midwife each time?	☐	☐	☐	☐	☐	

The questions below concern pregnancy check-ups with a midwife. If you saw more than one midwife, we ask that you give your overall impression.

	Not at all	To a small extent	To some extent	To a large extent	To a very large extent
12. Did the midwife treat you politely and with respect?	☐	☐	☐	☐	☐
13. Did the midwife spend enough time at the visits?	☐	☐	☐	☐	☐
14. Did you find that the midwife was open to your questions?	☐	☐	☐	☐	☐
15. Did you find that the midwife cared about you?	☐	☐	☐	☐	☐
16. Did you have confidence in the midwife's professional competence?	☐	☐	☐	☐	☐

17. Did you have pregnancy check-ups by a general practitioner?

☐ Yes → go to question 18 below

☐ No → go to question 26 on page 4

Pregnancy check-ups by a general practitioner

	1	2	3	4 or more
18. From how many different doctors did you receive pregnancy check-ups?	☐	☐	☐	☐

	Not at all	To a small extent	To some extent	To a large extent	To a very large extent	Not applicable
19. Was it easy to get an appointment with your general practitioner if you wanted to?	☐	☐	☐	☐	☐	☐
20. Was it important to you to get appointments from the same doctor each time?	☐	☐	☐	☐	☐	

The questions below concern pregnancy check-ups from your general practitioner. If you saw more than one doctor we ask that you give your overall impression.

	Not at all	To a small extent	To some extent	To a large extent	To a very large extent
21. Did the general practitioner treat you politely and with respect?	☐	☐	☐	☐	☐
22. Did the doctor spend enough time at the visits?	☐	☐	☐	☐	☐
23. Did you find that the general practitioner was open to your questions?	☐	☐	☐	☐	☐
24. Did you find that the general practitioner cared about you?	☐	☐	☐	☐	☐
25. Did you have confidence in the general practitioner's professional competence?	☐	☐	☐	☐	☐

Information at pregnancy check-ups

On the questions below we ask that you give your overall impression of the information you received at pregnancy check-ups by the midwife/general practitioner.

Did you receive sufficient information about:	Not at all	To a small extent	To some extent	To a large extent	To a very large extent	Not applicable
26. Your physical health during the pregnancy?	☐	☐	☐	☐	☐	☐
27. Possible mood changes during the pregnancy?	☐	☐	☐	☐	☐	☐
28. How the baby was developing?	☐	☐	☐	☐	☐	☐
29. What you could expect regarding birth?	☐	☐	☐	☐	☐	☐
30. Options for pain relief during birth	☐	☐	☐	☐	☐	☐
31. Post-natal period (for instance breast feeding, nutrition, care for the child)?	☐	☐	☐	☐	☐	☐

	Not at all	To a small extent	To some extent	To a large extent	To a very large extent	Not applicable
32. Did you experience that health personnel gave you conflicting information?	☐	☐	☐	☐	☐	☐

Ultrasound scan

We remind you that this questionnaire only concerns public services.

	1	2	3	4	5 or more	Not applicable
33. How many ultrasound scans did you have (including the routine ultrasound)?	☐	☐	☐	☐	☐	☐

	Yes	No, too few	No, too many	Not applicable
34. Do you think this was an appropriate number of ultrasounds?	☐	☐	☐	☐

	Not at all	To a small extent	To some extent	To a large extent	To a very large extent	Not applicable
35. Did you receive sufficient information concerning the ultrasound?	☐	☐	☐	☐	☐	☐
36. Were you happy with the midwife/doctor who performed the ultrasound?	☐	☐	☐	☐	☐	☐

Organization and availability

	Not at all	To a small extent	To some extent	To a large extent	To a very large extent	Not sure/ not applicable
37. Did you find that the public services you used during pregnancy were well organized?	☐	☐	☐	☐	☐	☐
38. Did you find that the midwife and general practitioner cooperated well?	☐	☐	☐	☐	☐	☐
39. Did you find that the midwife and the hospital cooperated well?	☐	☐	☐	☐	☐	☐
40. Did you find that the general practitioner and the hospital cooperated well?	☐	☐	☐	☐	☐	☐

Other considerations

	Not at all	To a small extent	To some extent	To a large extent	To a very large extent
41. All in all, were the public services you used during pregnancy satisfactory?	☐	☐	☐	☐	☐

	Much worse than expected	Somewhat worse than expected	As expected	Somewhat better than expected	Much better than expected
42. All in all, were the public services you used during pregnancy what you expected?	☐	☐	☐	☐	☐

	Not at all	To a small extent	To some extent	To a large extent	To a very large extent
43. Are you of the opinion that you were in any way given incorrect treatment (according to your own judgement)?	☐	☐	☐	☐	☐

PART B: THE BIRTH

44. Where did you give birth?

Delivery ward at a hospital	Birth centre, midwifery led (fødestue)	Planned home birth	Birth outside of hospital (not planned)
☐	☐	☐	☐

45. Did you give birth to one or more children?

One child	Twins	Triplets or more
☐	☐	☐

46. How many weeks into the pregnancy were you when the child/children were born?

Less than 28 weeks	28 – 32 weeks	33 - 36 weeks	37 – 41 weeks	42 weeks or more	Not sure
☐	☐	☐	☐	☐	☐

47. What was the child's birth weight?
If you gave birth to twins or more, please answer according to the child that was born first.

Under 1000 grams	1000 - 1500 grams	1501 - 2500 grams	2501 - 4500 grams	Over 4500 grams
☐	☐	☐	☐	☐

48. How did you give birth?

Normal birth	Forceps/ ventouse	Breech birth	Caesarean section
☐	☐	☐	☐

49. Did you have a hospital stay at the maternity ward/ delivery room connected to the birth?

☐ Yes → go to question 50 below

☐ No → go to question 71 on page 8

The stay at the delivery ward

This part concerns your stay at the delivery ward for the birth. Please also answer if you gave birth at a birth centre (fødestue).

	Not at all	To a small extent	To some extent	To a large extent	To a very large extent	Not applicable
50. Were you taken seriously by the health personnel at the delivery ward when you called to say the labour/birth had started?	☐	☐	☐	☐	☐	☐
51. Were you received well when you arrived at the delivery ward?	☐	☐	☐	☐	☐	

Health personnel at the delivery ward

	Not at all	To a small extent	To some extent	To a large extent	To a very large extent
52. Were you treated politely and with respect by the health personnel at the delivery ward?	☐	☐	☐	☐	☐
53. Did the health personnel have time for you when you needed it?	☐	☐	☐	☐	☐
54. Did you find that the health personnel were open to your questions?	☐	☐	☐	☐	☐
55. Did you find that the health personnel cared about you?	☐	☐	☐	☐	☐
56. Did you have confidence in the health personnel's professional competence?	☐	☐	☐	☐	☐

Information at the delivery ward

	Not at all	To a small extent	To some extent	To a large extent	To a very large extent
57. Did you receive sufficient information during your stay at the delivery ward?	☐	☐	☐	☐	☐
58. Did you experience that health personnel gave you conflicting information?	☐	☐	☐	☐	☐

Organization and involvement at the delivery ward

	Not at all	To a small extent	To some extent	To a large extent	To a very large extent	Not sure/ not applicable
59. Did you find that the services you received during your stay at the delivery ward were well-organized?	☐	☐	☐	☐	☐	☐
60. Did you find that the health personnel cooperated well during the birth?	☐	☐	☐	☐	☐	☐
61. Did you receive information about who had the main responsibility for you?	☐	☐	☐	☐	☐	
62. Did you wish to be involved in the decisions concerning your birth?	☐	☐	☐	☐	☐	☐
63. Were you involved in decisions concerning your birth?	☐	☐	☐	☐	☐	☐
64. Did you receive sufficient pain relief during labour?	☐	☐	☐	☐	☐	☐

Partner

	Not at all	To a small extent	To some extent	To a large extent	To a very large extent	Not applicable
65. Was your partner received well by the health personnel at the delivery ward?	☐	☐	☐	☐	☐	☐
66. Were things arranged so that your partner could be present if you both so wished?	☐	☐	☐	☐	☐	☐

Other considerations

	Not at all	To a small extent	To some extent	To a large extent	To a very large extent
67. All in all, were you satisfied with the services you received during your stay at the delivery ward?	☐	☐	☐	☐	☐

	Much worse than expected	Somewhat worse than expected	As expected	Somewhat better than expected	Much better than expected
68. All in all, were the services you received during your stay at the delivery ward what you expected?	☐	☐	☐	☐	☐

	Not at all	To a small extent	To some extent	To a large extent	To a very large extent
69. Are you of the opinion that your child was in any way given incorrect treatment (according to your own judgement)?	☐	☐	☐	☐	☐
70. Are you of the opinion that you were in any way given incorrect treatment (according to your own judgement)?	☐	☐	☐	☐	☐

71. Did you stay in the maternity ward/ patient hotel (barselhotell)/ birth centre (fødestue) after giving birth?

☐ Yes → go to question 72 on page 9

☐ No → go to question 102 on page 12

Post-natal stay

This part concerns your stay at the maternity ward, patient hotel (barselhotell) or birth centre (fødestue) after the birth. If you have experiences from more than one place, we ask you to give an overall impression.

	Less than 24 hours	1 - 2 days	3 - 4 days	5 - 6 days	7 days or more
72. How long did you stay after birth?	☐	☐	☐	☐	☐

	Yes	No, too short	No, too long
73. Do you think the lenght of stay was appropriate?	☐	☐	☐

	Single patient room in maternity ward	Multiple-bed room in maternity ward	Family room in maternity ward	Patient hotel (barsel-hotell)	Birth centre (fødestue)	Other
74. Where did you spend most of the post-natal period?	☐	☐	☐	☐	☐	☐

Health personnel during the post-natal stay

	Not at all	To a small extent	To some extent	To a large extent	To a very large extent
75. Were you treated politely and with respect by the health personnel during the post-natal stay?	☐	☐	☐	☐	☐
76. Did the health care personnel have time for you when you needed it?	☐	☐	☐	☐	☐
77. Did you find that the health personnel were open to your questions?	☐	☐	☐	☐	☐
78. Did you find that the health personnel cared about you and your child?	☐	☐	☐	☐	☐
79. Did you have confidence in the health personnel's professional competence?	☐	☐	☐	☐	☐

Information during the post-natal stay

Did you receive sufficient information about:

	Not at all	To a small extent	To some extent	To a large extent	To a very large extent	Not applicable
80. Your physical health after giving birth?	☐	☐	☐	☐	☐	☐
81. Any possible mood changes after birth?	☐	☐	☐	☐	☐	☐
82. Breast feeding or other ways of feeding the child?	☐	☐	☐	☐	☐	☐
83. Child care?	☐	☐	☐	☐	☐	☐

	Not at all	To a small extent	To some extent	To a large extent	To a very large extent
84. Did you experience that health personnel gave you conflicting information?	☐	☐	☐	☐	☐

Guidance and support during the post-natal stay

	Not at all	To a small extent	To some extent	To a large extent	To a very large extent	Not applicable
85. Did you receive sufficient guidance on breast feeding or other ways of feeding the child during the post-natal stay?	☐	☐	☐	☐	☐	☐
86. Did you receive sufficient guidance on child care during the post-natal stay?	☐	☐	☐	☐	☐	☐

Organization during the post-natal stay

	Not at all	To a small extent	To some extent	To a large extent	To a very large extent	Not sure/ not applicable
87. Did you find that the services you received during your post-natal stay were well organized?	☐	☐	☐	☐	☐	☐
88. Did you find that the health personnel cooperated well during your post-natal stay?	☐	☐	☐	☐	☐	☐
89. Did you receive information about who had the main responsibility for you?	☐	☐	☐	☐	☐	
90. Were things arranged so that you could get enough peace and rest?	☐	☐	☐	☐	☐	

Partner

	Not at all	To a small extent	To some extent	To a large extent	To a very large extent	Not applicable
91. Was your partner received well by the health personnel during the post-natal stay?	☐	☐	☐	☐	☐	☐
92. Were things arranged so that your partner could be present if you both so wished?	☐	☐	☐	☐	☐	☐

Going home

	Yes, an individual consultation	Yes, a group consultation	No			
93. Did you have a consultation with a midwife before going home (check-out consultation/ utreisesamtale)?	☐	☐	☐			

	No benefit	Small benefit	Some benefit	Large benefit	Very large benefit	Did not have such a consultation
94. What benefit did you have from this consultation?	☐	☐	☐	☐	☐	☐

	Yes	No				Not sure
95. Were you informed about where you could inquire in case of any questions after you got home?	☐	☐				☐

	Not at all	To a small extent	To some extent	To a large extent	To a very large extent	Did not initiate contact
96. Were the contact people easy to get in touch with?	☐	☐	☐	☐	☐	☐
97. Were your questions sufficiently answered if you contacted someone?	☐	☐	☐	☐	☐	☐

Other considerations

	Not at all	To a small extent	To some extent	To a large extent	To a very large extent
98. All in all, were you satisfied with the services you received during your post-natal stay?	☐	☐	☐	☐	☐

	Much worse than expected	Somewhat worse than expected	As expected	Somewhat better than expected	Much better than expected
99. All in all, were the services you received during your post-natal stay what you expected?	☐	☐	☐	☐	☐

	Not at all	To a small extent	To some extent	To a large extent	To a very large extent
100. Are you of the opinion that your child was in any way given incorrect treatment during the post-natal stay (according to your own judgement)?	☐	☐	☐	☐	☐
101. Are you of the opinion that you were in any way given incorrect treatment during the post-natal stay (according to your own judgement)?	☐	☐	☐	☐	☐

102. Have you had any contact with the public health clinic (helsestasjon) since the birth (i.e. by telephone, at check ups/ vaccinations, had home visits)?

☐ Yes → go to question 103 below

☐ No → go to question 135 on page 14

PART C: CONTACT WITH HEALTH CLINIC (HELSESTASJON) AFTER GIVING BIRTH

This part concerns contact with the public health clinic in the period after the birth and up to now. If you have experiences from more than one health clinic please answer based on an overall impression.

Contact with the public health clinic (helsestasjon)

103. Have you been offered home visits from the health clinic?	Yes ☐	No ☐				
104. What benefit did you have from the home visit from the health clinic?	No benefit ☐	Small benefit ☐	Some benefit ☐	Large benefit ☐	Very large benefit ☐	Did not receive any home visit ☐

105. How long after the birth were you at the first check-up at the health clinic?	Less than 2 weeks after the birth ☐	3-6 weeks after the birth ☐	7-9 weeks after the birth ☐	10 weeks or more after the birth ☐		Not applicable ☐
106. Did you have to initiate contact yourself to get an appointment for the first check-up at the health clinic?	Yes ☐	No ☐				Not applicable ☐
107. Would you have liked the first check-up to have been earlier than it was?	Yes ☐	No ☐				Not applicable ☐
108. How many check-ups have you had at the health clinic up to now?	None ☐	1 ☐	2 ☐	3 - 5 ☐	6 or more ☐	Not applicable ☐
109. Do you think this has been an appropriate number of check-ups?	Yes ☐	No, too few ☐	No, too many ☐			Not applicable ☐

110. What benefit did you have from the child's six-week check-up?	No benefit ☐	Small benefit ☐	Some benefit ☐	Large benefit ☐	Very large benefit ☐	Did not have this check-up ☐
111. What benefit did you have from your own six-week check-up (post-check-up)?	☐	☐	☐	☐	☐	☐

Health clinic staff

	Not at all	To a small extent	To some extent	To a large extent	To a very large extent	Not applicable
112. Is it important to you to get appointments with the same person at each check-up?	☐	☐	☐	☐	☐	☐

	Not at all	To a small extent	To some extent	To a large extent	To a very large extent	Not applicable
113. Did you find that the staff members at the health clinic were informed about your birth?	☐	☐	☐	☐	☐	☐
114. Are you treated politely and with respect by the staff?	☐	☐	☐	☐	☐	☐
115. Does the staff spend enough time on the check-ups?	☐	☐	☐	☐	☐	☐
116. Do you find that the staff is open to your questions?	☐	☐	☐	☐	☐	☐
117. Do you find that the staff cares about you and your child?	☐	☐	☐	☐	☐	☐
118. Do you have confidence in the staff's professional competence?	☐	☐	☐	☐	☐	☐

Information from the health clinic

On the questions below we ask that you give your overall impression of the information you received from the health clinic.

Have you received sufficient information about:

	Not at all	To a small extent	To some extent	To a large extent	To a very large extent	Not applicable
119. Services at the health clinic?	☐	☐	☐	☐	☐	☐
120. Your physical health after giving birth?	☐	☐	☐	☐	☐	☐
121. Possible mood changes after birth?	☐	☐	☐	☐	☐	☐
122. The child's development and health?	☐	☐	☐	☐	☐	☐
123. Vaccines for the child?	☐	☐	☐	☐	☐	☐
124. Breast feeding or other ways of feeding the child?	☐	☐	☐	☐	☐	☐
125. Child care?	☐	☐	☐	☐	☐	☐

	Not at all	To a small extent	To some extent	To a large extent	To a very large extent
126. Have you experienced that the staff have been giving you conflicting information?	☐	☐	☐	☐	☐

Organization and availibility of health clinic

	Not at all	To a small extent	To some extent	To a large extent	To a very large extent	Not sure/ not applicable
127. Do you find that the care you receive at the health clinic is well organized?	☐	☐	☐	☐	☐	☐
128. Has it been easy to make a check-up appointment if you so wished?	☐	☐	☐	☐	☐	☐

	Yes	No	Not sure
129. Have you been informed of where you can inquire if you have any questions between the check-ups?	☐	☐	☐

	Not at all	To a small extent	To some extent	To a large extent	To a very large extent	Did not initiate contact
130. Have the contact people been easy to get in touch with?	☐	☐	☐	☐	☐	☐
131. Have your questions been sufficiently answered if you have contacted someone?	☐	☐	☐	☐	☐	☐

Other considerations

	Not at all	To a small extent	To some extent	To a large extent	To a very large extent
132. All in all, are you satisfied with the services you have received at the health clinic?	☐	☐	☐	☐	☐

	Much worse than expected	Somewhat worse than expected	As expected	Somewhat better than expected	Much better than expected
133. All in all, are the services you have received at the health clinic what you expected?	☐	☐	☐	☐	☐

	Not at all	To a small extent	To some extent	To a large extent	To a very large extent
134. Are you of the opinion that your child was in any way given incorrect treatment at the health clinic (according to your own judgement)?	☐	☐	☐	☐	☐

	Yes	No
135. Have you had any contact with a post-natal/ breast feeding outpatient clinic since the birth?	☐	☐

PART D: BACKGROUND QUESTIONS

136. What is your age?

Years ☐

137. Are you married or cohabiting?

Yes, married	Yes, cohabiting	No
☐	☐	☐

138. What is your highest completed level of education?

Primary/ lower secondary school	Upper secondary school	College/ university (1–4 years)	College/ university (more than 4 years)
☐	☐	☐	☐

139. What is your everyday activity when you are not on maternity leave? *Select only one answer.*

Employed	On sick leave, on disability or work assessment allowance	Student	Housekeeping (in your home)	Unemployed	Other
☐	☐	☐	☐	☐	☐

140. Overall, would you say your health is...

Excellent	Very good	Good	Fair	Poor
☐	☐	☐	☐	☐

141. How many times have you given birth in the past?

Never	1	2	3	4 or more
☐	☐	☐	☐	☐

142. Did your caesarian wound get infected (e.g. inflammation/pus in the operation wound)?

Yes, at the hospital	Yes, after I got home	No	Did not have a caesarian section
☐	☐	☐	☐

143. Where was the infection treated?

At my general practitioner's office	At the health clinic (helsestasjon)	At the hospital where I gave birth	At another hospital	At the casualty clinic (legevakten)	Other	The infection was not treated	Did not get an infection	I did not have a caesarian section
☐	☐	☐	☐	☐	☐	☐	☐	☐

144. Do you feel that the infection was taken care of in a satisfactory way?

Yes	No	Did not initiate contact	Did not get an infection	I did not have a caesarian section
☐	☐	☐	☐	☐

If you wish, you may write more about your experiences here:

Thank you very much for taking the time to answer!

kunnskapssenteret
